# Divorce Rates Better Predict Population‐Level Reproductive Success in Little Penguins Than Foraging Behaviour or Environmental Factors

**DOI:** 10.1002/ece3.70787

**Published:** 2025-01-11

**Authors:** Matthew D. Simpson, Ashton L. Dickerson, Andre Chiaradia, Lloyd Davis, Richard D. Reina

**Affiliations:** ^1^ School of Biological Sciences Monash University Clayton Victoria Australia; ^2^ Leibniz‐Institute of Freshwater Ecology and Inland Fisheries Berlin Germany; ^3^ Conservation Department Phillip Island Nature Parks Cowes Victoria Australia; ^4^ Centre for Science Communication University of Otago Dunedin New Zealand

**Keywords:** divorce, *Eudyptula minor*, foraging, little penguins, marine environmental conditions, reproductive success

## Abstract

Understanding the relative contributions of environmental, behavioural and social factors to reproductive success is crucial for predicting population dynamics of seabirds. However, these factors are often studied in isolation, limiting our ability to evaluate their combined influence. This study investigates how marine environmental variables, foraging behaviour and social factors (divorce), influence reproductive success in little penguins ( *Eudyptula minor* ) over 13 breeding seasons. By examining these factors together, we aimed to identify which is the most reliable predictor of population‐level reproductive success. We found that divorce rate was the most consistent predictor of reproductive success, with lower annual rates of divorce preceding the breeding season associated with higher hatching and fledging success. Foraging trip duration also influenced reproductive success, but in contrasting ways: Longer trips during egg incubation were linked with increased hatching success, while shorter trips after hatching led to higher fledging success. Marine environmental conditions had unexpected effects, with a lower Southern Oscillation Index (SOI) correlating with improved hatching and fledging success, in contrast to previous research, while sea surface temperature (SST) had no significant effect on reproductive success. This highlights the complexity of seabird breeding responses to large‐scale oceanographic indices, suggesting SOI and SST are generally less reliable measures to use as indicators of reproductive success. Our results suggest that divorce rate could serve as a valuable, noninvasive index of reproductive success in seabirds.

## Graphical abstract

This study explored factors influencing reproductive success in little penguins, focusing on environmental conditions, foraging behaviour and the social factor divorce. Divorce rate emerged as the strongest predictor of breeding success, with lower divorce rates linked to higher hatching and fledging success. In contrast, foraging duration and environmental conditions had a limited impact, suggesting that divorce rate could serve as a valuable indicator for predicting population‐level reproductive outcomes in seabirds.

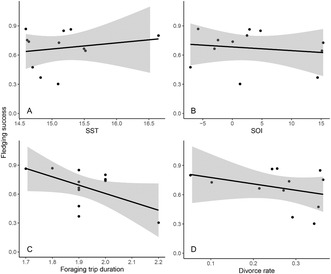

## 1 Introduction

Understanding the factors affecting reproductive success is critical for predicting population trends, yet the combined effects of environmental variability and individual behaviour remain poorly understood. Most studies focus on either ecological or behavioural factors (e.g., Becker, Peery, and Beissinger 2007; Boersma and Rebstock 2009; Nisbet and Dann 2009), but few have examined their concurrent influence on reproductive outcomes. This gap limits our ability to predict how dynamic environmental conditions and individual behavioural strategies interact to shape population‐level reproductive success and to identify the most reliable predictors. Seabirds provide an ideal system for investigating these trade‐offs, because as central‐place foragers with high parental investment, seabirds must coordinate foraging in unpredictable marine environments with offspring care (Schreiber and Burger 2001; Ichii et al. 2007; Genovart et al. 2013; Afán et al. 2015). Their breeding strategies offer valuable information on how ecological and behavioural factors shape reproductive outcomes and reveal the key drivers of population‐level reproductive success in dynamic environments.

Seabird reproductive success is constrained by variability in ocean productivity at global, regional and local scales (Berlincourt and Arnould 2015; Peck et al. 2004; Lescroël et al. 2009; Becker, Peery, and Beissinger 2007; Kowalczyk et al. 2015). At the global scale, the El Niño cycle, a natural climate pattern characterised by unusually warm ocean temperatures in the equatorial Pacific, can cause reduced prey abundance in ocean basins such as the Western area of the South Pacific Ocean (Ancona, Calixto‐Albarran, and Drummond 2012; Genovart et al. 2013). These events can be predicted using the Southern Oscillation Index (SOI), which measures air pressure differences between the western and eastern Pacific to indicate the strength and phase of El Niño. Regionally, changes in sea surface temperature (SST) can result from upwelling and reduce the input of nutrients supplying the growth of prey populations (Peck et al. 2004; Weeks, Steinberg, and Congdon 2013). Higher SSTs can reduce prey availability for seabirds due to decreased productivity at lower trophic levels and increased movement of fish (Peck et al. 2004; Erwin and Congdon 2007; Afán et al. 2015). Locally, spatial distribution of prey can be altered through stratification of the water column and the presence of a thermocline can act as a thermal barrier to prey (Pelletier et al. 2012; Garthe et al. 2014; Meyer et al. 2020).

Foraging behaviour in seabirds is intrinsically linked to environmental conditions, with prey availability influencing the effort required to find sufficient food. Most seabirds are central‐place foragers obliged to return to land to care for their offspring, restricting their foraging range and duration (Schreiber and Burger 2001; Ichii et al. 2007; Orians and Pearson 1979). Unsuccessful foraging can be expensive for seabirds, as it expends energy and time (days or even weeks) without securing resources, potentially leading to poorer body condition (Boggs 1992; Chivers et al. 2012). This can reduce reproductive success through lower chick survival, delayed fledging or aborted breeding attempts (Chiaradia and Nisbet 2006; Joly et al. 2023). Moreover, repeated foraging failures may increase adult mortality rates (Harding et al. 2011). The distance that individuals must travel to access suitable foraging zones is particularly important, because birds that travel further to access a foraging zone must increase time away from their offspring (Terauds and Gales 2006; Boersma and Rebstock 2009; Saraux et al. 2011a; Jakubas et al. 2013) and their reproductive success can be lower when chicks are forced to wait for long periods between meals (Chiaradia and Nisbet 2006). Additionally, the incubating partner may be forced to abandon the nest in order to replenish their own fat stores if they are left alone too long (Olsson 1997). Consequently, foraging trips durations may be a strong determinant of reproductive success.

Divorce rates represent a critical factor related to reproductive success, alongside environmental and foraging constraints. Divorce refers to the dissolution of a breeding pair bond, leading individuals to seek new mates (Ens, Safriel, and Harris 1993; Gousy‐Leblanc et al. 2023). This behaviour is influenced by various factors, including environmental stressors and reproductive failure, which can impact the stability of pair bonds. For example, pairs are more likely to divorce when they fail to produce fledglings or when they face challenging environmental conditions that can additionally cause asynchronous arrival times at breeding sites (Setiawan et al. 2005; Gunnarsson et al. 2004; Afán et al. 2015; Ventura et al. 2021). The drivers of divorce are often overlapping and vary between species, highlighting the complexity of this behaviour (Choudhury 1995; Setiawan et al. 2005). While maintaining a pair bond can be less costly than finding a new mate, especially in species with low divorce rates such as seabirds (Fowler 1995; van de Pol and Verhulst 2006), divorce may enhance long‐term reproductive success by enabling individuals to find more compatible or higher quality mates (Choudhury 1995; Gousy‐Leblanc et al. 2023). However, in years of with low re‐pairing rates (high divorce rates), the population as a whole may experience reduced reproductive output for that breeding season due to the time spent in mate‐searching and courtship, delaying reproduction or even preventing it altogether (Gousy‐Leblanc et al. 2023; Verboven and Verhulst 1996; Moody et al. 2005). Furthermore, this may force parents to forage for their chicks during times of poorer food availability because of the delay in beginning reproduction caused by having to spend time finding a new mate (Verboven and Verhulst 1996; Moody et al. 2005). Consequently, divorce rates at the beginning of a breeding season may serve as an important indicator of reproductive success, particularly when environmental conditions limit resources and impact both foraging behaviour and the ability to maintain pair bonds (Gousy‐Leblanc et al. 2023).

In this study, we investigated the effects of environmental conditions, foraging behaviour and divorce rates on reproductive success in a colony of little penguins ( *Eudyptula minor* ) over 13 breeding seasons. Little penguins are a useful system for this research due to their small foraging range (typically < 20 km) and the sensitivity of their reproductive success to environmental changes (Hoskins et al. 2008; Pelletier et al. 2014; Saraux and Chiaradia 2022). Changes in foraging conditions, including variations in SST and the SOI, have been shown to significantly impact their reproductive outcomes (Chiaradia and Nisbet 2006; Saraux et al. 2011b). Furthermore, little penguins benefit from maintaining pair bonds, with pair longevity related to increased reproductive success (Nisbet and Dann 2009). By investigating these three factors in tandem, our aim was to identify which of these variables, environmental conditions, foraging behaviour or divorce rates, emerged as the most consistent predictor of population‐level reproductive success. We predicted that years of more favourable environmental conditions (higher SOI values, lower SST) would be associated with shorter foraging trip durations and higher reproductive success, while lower divorce rates would also contribute to greater reproductive success. This approach offered a more integrated understanding of the ecological and behavioural drivers of reproductive success and aimed to provide a more reliable index for monitoring reproductive trends in seabird populations.

## 2 Materials and Methods

### 2.1 Study Site and Monitoring Protocols

We investigated little penguins at the Penguin Parade site of the megacolony located at the western end of Phillip Island, Victoria, Australia (38°15′ S, 143°30′ E) with a population size of about 28,000 to 32,000 penguins (Sutherland and Dann 2012). Data from a study site of approximately 100 artificial nest boxes within the larger Summerland Peninsula were used over 13 breeding seasons (August–February; 2000–2012), which provides a good proxy of the whole population (Sutherland and Dann 2014). Each year, ~70% of nest boxes were occupied (average number of pairs monitored each year 71.5, range 50–98). All penguins within the study colony were permanently identified with unique‐numbered electronic transponders (Allflex Australia Pty Ltd., Capabala, Queensland) that were injected subcutaneously between the scapula (Chiaradia and Kerry 1999). The presence of adults within the study area was detected using a purpose‐built handheld transponder reader and to minimise disturbance to the birds the reader was passed along the exterior surface of the walls of the nest boxes. Once birds began pairing and courtship was observed, this was considered the beginning of the breeding season, and data collection began (Chiaradia and Kerry 1999). During the breeding season, nest attendance was recorded three times a week and nests were checked for chicks and the different stages of breeding were recorded (Table S1 for definitions of breeding stages).

As measures for the population reproductive success (summarised in Table S1), we recorded the average number of eggs per breeding pair for the season, hatching success (proportion of eggs that hatched), fledging success (proportion of chicks that fledged) and the average number of fledglings per pair for the season. Chicks were considered to have successfully fledged upon reaching > 45 days of age and being fully feathered since the last encounter (Chiaradia and Nisbet 2006). Monitoring within a season ended once all nest boxes were empty of pairs or chicks.

### 2.2 Environmental Variables

For global marine conditions, average values of monthly SOI from 2000 to 2012 from September to January (corresponding to the breeding season) were obtained from the Bureau of Meteorology, Australia (http://bom.gov.au). In the southern Australian climate, positive values indicate wetter conditions (moving towards La Niña), with cooler daytime temperatures, while negative values indicate drier and warmer conditions (moving towards El Niño).

Regional conditions were indicated by data for SST for the waters off Phillip Island, obtained from the US National Oceanic & Atmospheric Administration website (http://www.esrl.noaa.gov). In situ and satellite SSTs along with SSTs simulated by sea ice cover (Reynolds et al. 2002), were used to conduct optimum interpolation analysis (NOAA OI SST V2) to calculate the SSTs used in this study (giving regional conditions). Weekly SST data were averaged from September to January in the years 2000 to 2012, and averaged for a one‐degree grid, 38 °S–40° S × 143° E–145° E (Cullen et al. 2009).

Data for local conditions were inferred from delta temperature (Δ*T*) using temperature profiles from Bluelink Ocean Data Assimilation (BODAS, Oke et al. 2007). To calculate Δ*T*, the surface temperature (0 m) was taken from SST and temperature at 50 m was subtracted as a measure of stratification in the water column (Oke et al. 2005, 2007). Data were weekly means in the 1° × 1° box defined by −38° S –39° S × 143.5° E—145.5° E from September 2000 to December 2012.

### 2.3 Foraging Trips

Durations of foraging trips of individual penguins were determined using an Automated Penguin Monitoring System (APMS), developed by the Australian Antarctic Division (see Chiaradia and Kerry 1999 for further details) and modified by Kean Electronics (https://www.kean.com.au/) that monitored colony attendance by birds. The APMS was located at the main colony entrance and when a bird crossed the weighbridge the reader recorded the transponder number, time and date (Kerry, Clarke, and Else 1993). To track the arrival and departure of individuals from the colony, the APMS recorded continuously over 13 breeding years from 2000 to 2012. Using the data from the weighbridge, we determined the duration of their trips in days. Only birds that had successfully fledged chicks were used when determining foraging trips, which vary over the postguard stage and are critical for fledging success (Saraux et al. 2011b).

### 2.4 Divorce Rate

Divorce rate was calculated at a population level for each breeding season using the data from nest box monitoring. We crossreferenced the presence of individual penguins with their partners from the previous season to categorise relationships. The individual was classified as divorced if its partner from the immediately prior breeding season partner reappeared in the colony but was paired with a new mate in the current season in which its reproductive success was determined. The breeding individual was classified as widowed if its partner from the previous breeding season partner was not observed during the current season. Although little penguins may skip a breeding season or breed outside the study site, these are infrequent events (Joly et al. 2023). Any bias in the calculation from the difference between observed and expected values would be a systematic error repeating over years, without interfering with the reliability and validity of results. As divorce rate could only be calculated by comparing pairings from previous years, the breeding season of 2000 (the starting year in this study) did not have a divorce rate assigned to it, meaning that this year could not be used in the models (resulting in a final sample size of 12 breeding seasons). Within‐season divorce in the same season, following a breeding attempt with a new partner, is rare in little penguins and was not observed in the Phillip Island colony during this study.

### 2.5 Statistical Analysis

Four models were constructed to explore how behaviour and environment influences reproductive success. Response variables indicating reproductive success for each season were the average number of eggs laid per pair, hatching success (proportion of eggs hatched of eggs laid), fledging success (proportion of fledglings of chicks hatched) and the average number of fledged chicks per pair. Linear models were used on average number data while generalised linear models (GLMs) with a binomial distribution were used to analyse proportional data. Predictor variables considered were the environmental variables SST, SOI and Δ*T*, and the behavioural predictors mean foraging trip duration and divorce rate. Before building our models, we tested for collinearity between the predictors. Sea surface temperature was significantly higher with low Δ*T* (*R* = −0.91). As the R value exceeded the cut off 0.7 between these two variables, we excluded Δ*T* to avoid collinearity problems (Tabachnick and Fidell 2018). We removed Δ*T* instead of SST, as SST is known to have a strong effect on prey availability (Afán et al. 2015; Pelletier et al. 2014), while changes in Δ*T* can be mitigated with behavioural tactics (Meyer et al. 2020). The final predictor variables included for each model were SST, SOI, mean foraging trip duration and divorce rate.

Model selection was used to determine the strongest models, using conservative Akaike Information Criterion corrected for small sample sizes (AIC_c_) (Anderson and Burnham 2002). To assess the importance of factors across models, we performed model averaging using the MuMIn package in R (Burnham and Anderson 2002; Symonds and Moussalli 2011). All models with ΔAIC_c_ ≤ 6 were used in the model averaging. This threshold allows for the inclusion of models with substantial empirical support relative to the best‐performing model, particularly in complex ecological data sets where multiple plausible models may explain variation (Richards 2005). Narrower thresholds, such as ΔAIC_c_ < 2, risk excluding models that contribute valuable insights but perform slightly less well due to minor structural or stochastic differences. We used the conditional average method as this only averages parameters over models in which they appear (Grueber et al. 2011; Burnham and Anderson 2002). This approach provides unbiased estimates of effect sizes while retaining biological interpretability. If the top model was a subset of another retained model and did not provide a sufficient improvement in loglikelihood, these were deemed to contain uninformative parameters. We then excluded the competing model to avoid over‐weighting parameters during model averaging (Burnham and Anderson 2004; Arnold 2010; Grueber et al. 2011). All statistical analysis was conducted using R version 4.2.2 (R Core Team 2022).

## 3 Results

Measures of reproductive success varied across the monitoring period (Figure S1), as did the environmental marine conditions (SST average = 15.17, range = 14.60–16.65; SOI average = 3.77, range = −7.08—15.34), foraging trip duration (average = 1.91 days, range = 1.7–2.2 days) and divorce rate (average 0.26, range = 0.05–0.36) (Figure S2). Among these variables, divorce rate was significantly higher with decreasing SST (*R* = −0.69) (Table 1). No other variables used in the final models were significantly correlated.

**TABLE 1:** Spearman correlations between behavioural and environmental parameters measured at a little penguin colony at Phillip Island, Australia (*n* = 12 breeding seasons).

Parameter	Correlations			
	1.	2.	3.	4.
1. Divorce rate				
2. Foraging trip duration (days)	0.15			
3. Delta temperature (Δ*T*)	0.69*	−0.12		
4. SST	−0.69*	0.08	−0.91***	
5. SOI	−0.30	−0.05	−0.12	0.22

***< 0.001, *< 0.05.

### 3.1 Number of Eggs

**FIGURE 1:**
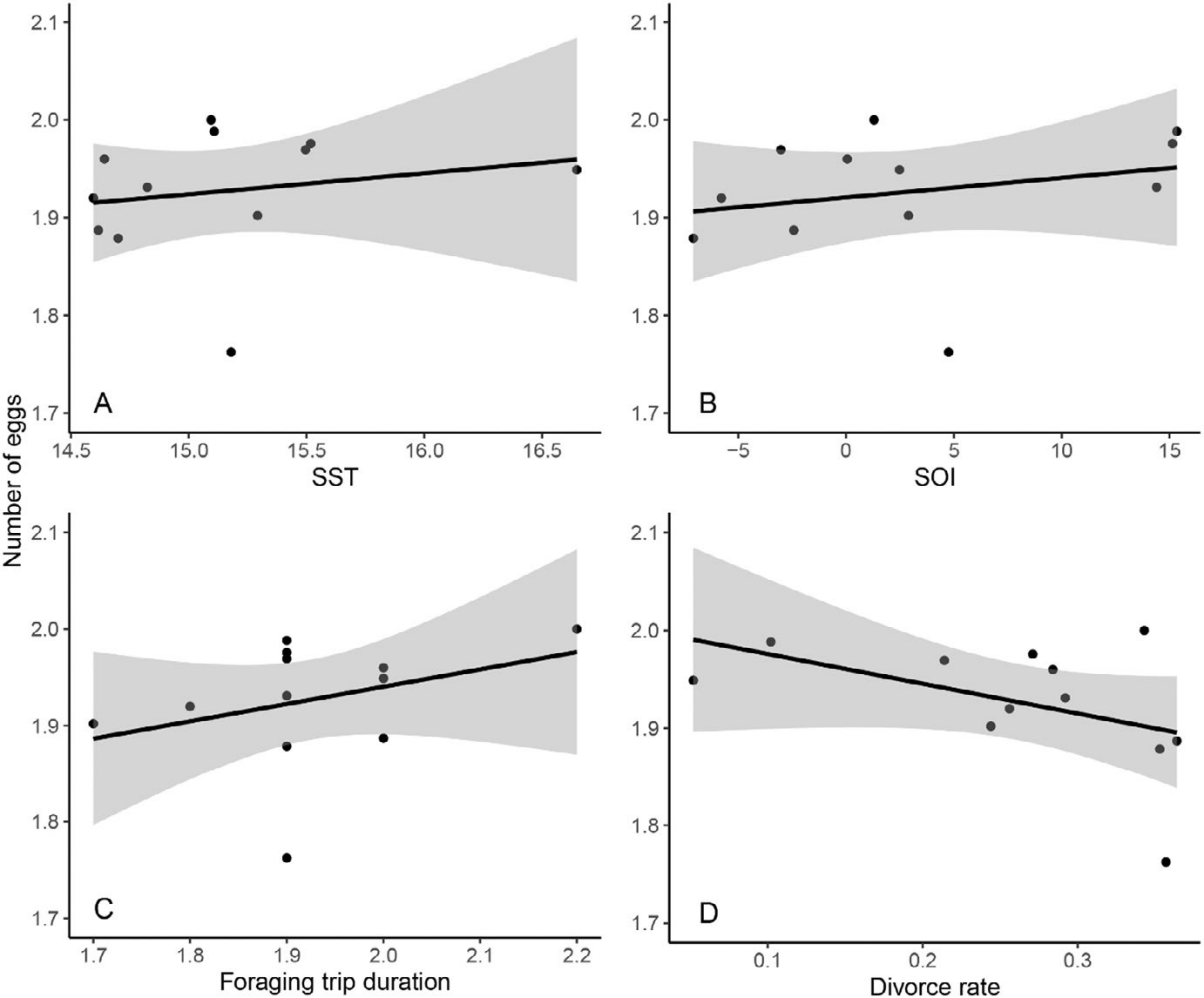
Average number of eggs laid by pairs each breeding season (2001–2012) in a population of little penguins, at Phillip Island, Australia, against predictor variables (A) SST, (B) SOI, (C) foraging trip duration (days) and (D) divorce rate.

Over the monitoring period the average number of eggs per pair each season remained consistent (average = 1.93, range = 1.76–2.00, Figure S1a), with nine best‐fit models (two were excluded due to uninformative parameters, Table S2). No environmental or behavioural measures significantly affected number of eggs laid by pairs (Table 2, Figure 1).

**TABLE 2:** Effect size estimate, standard error and confidence intervals produced by model averaging of environmental and behavioural predictor variables on the average number of eggs produced per pair in little penguins at Phillip Island, Australia (*N* = 12 breeding seasons).

Parameter	Estimate	Unconditional SE	Confidence interval	Relative importance
*Intercept*	1.873	0.269	(1.345, 2.400)	
Divorce rate	−0.302	0.211	(−0.716, 0.113)	0.30
Foraging trip duration (days)	0.180	0.160	(−0.177, 0.537)	0.15
SOI	0.002	0.003	(−0.004, 0.008)	0.14
SST	0.022	0.035	(−0.056, 0.099)	0.08

### 3.2 Hatching Success

**FIGURE 2:**
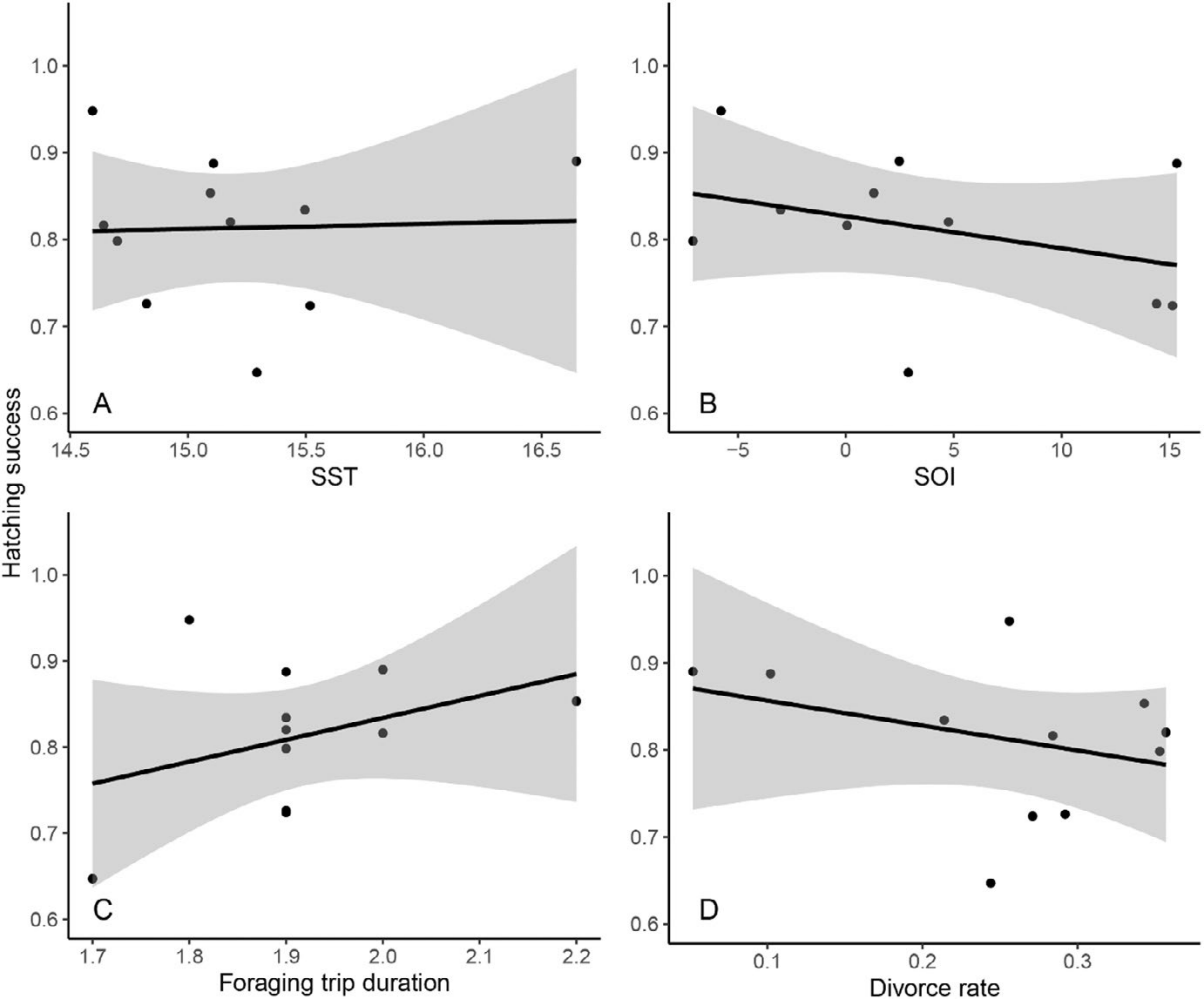
Hatching success (proportion of hatched eggs by number of eggs laid) each breeding season (2001–2012) in a population of little penguins, at Phillip Island, Australia, against predictor variables (A) SST, (B) SOI, (C) foraging trip duration (days) and (D) divorce rate.

The proportion of hatched eggs across seasons was on average 0.79 (range 0.59–0.95) (Figure S1b). Five models were best‐fit, with one removed for uninformative parameters (Table S3). In years of lower divorce rate and longer average foraging trip duration, the hatching success was significantly higher (Table 3, Figure 2). Years with lower SOI values had higher hatching success. Divorce rate had the highest relative importance (*R* = 1.00), followed by foraging trip duration (*R* = 0.92) then SOI (*R* = 0.78).

**TABLE 3:** Effect size estimate, standard error and confidence intervals produced by model averaging of environmental and behavioural predicator variables on proportion of hatching success in little penguins at Phillip Island, Australia (*N* = 12 breeding seasons).

Parameter	Estimate	Unconditional SE	Confidence interval	Relative importance
*Intercept*	0.187	2.383	(−4.484, 4.859)	
Divorce rate	−4.284	1.052	(−6.345, −2.223)	1.00
Foraging trip duration (days)	1.674	0.669	(0.362, 2.986)	0.92
SOI	−0.020	0.009	(−0.039, −0.002)	0.78
SST	−0.312	0.207	(−0.717, 0.093)	0.13

### 3.3 Fledging Success

**FIGURE 3:**
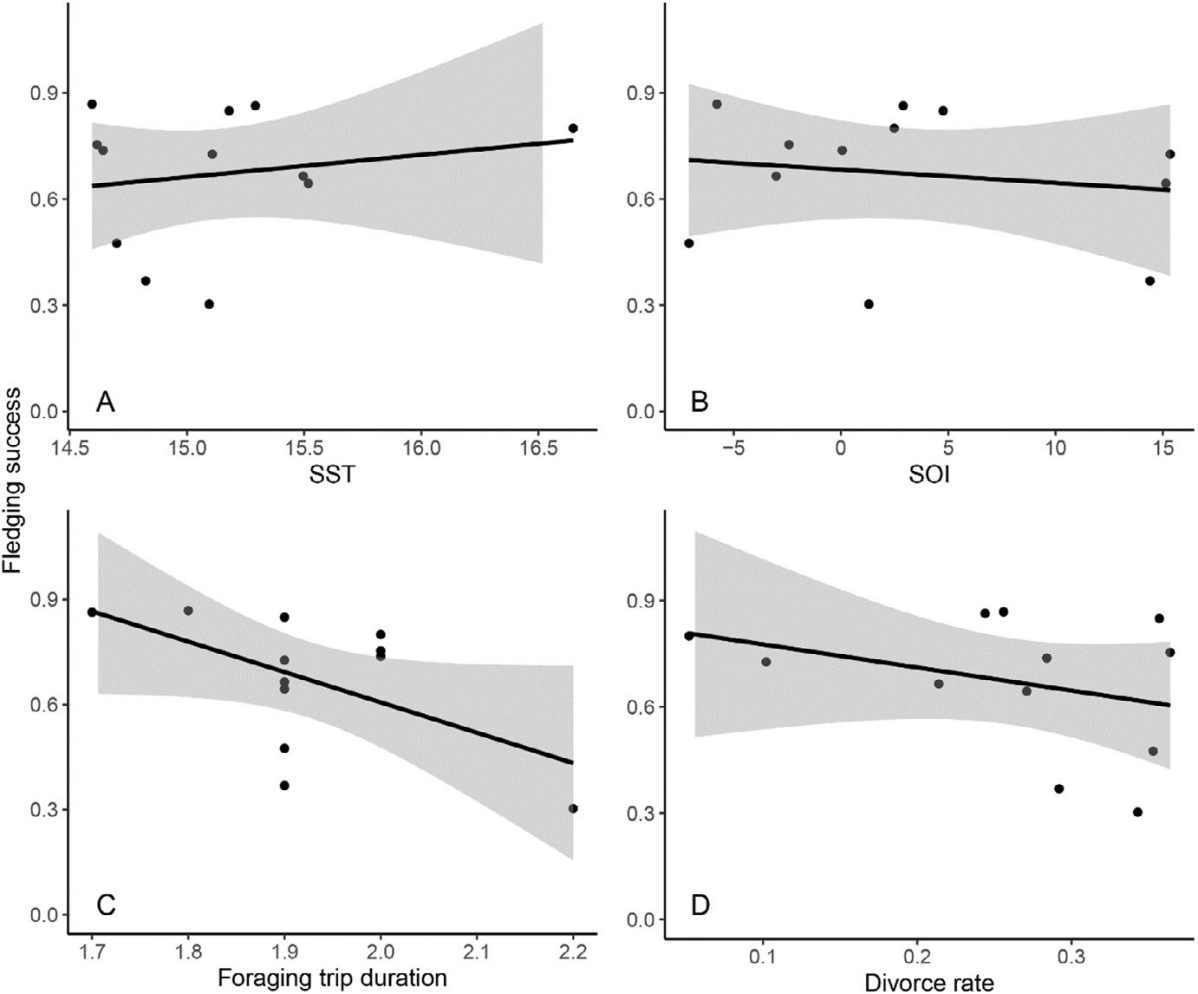
Fledging success (proportion of fledged chicks by number of chicks hatched) each breeding season (2001–2012) in a population of little penguins, at Phillip Island, Australia, against predictor variables (A) SST, (B) SOI, (C) foraging trip duration (days) and (D) divorce rate.

The proportion of successfully fledged chicks across seasons varied widely across the monitoring period, with an average of 0.67 (range 0.30–0.87, Figure S1c). Lower divorce rates, shorter average foraging trip durations and years of more negative SOI values significantly increased fledging success (Table 4, Figure 3), as found by two best‐fit models (Table S4). Divorce rate, foraging trip duration and SOI all had equally high relative importance (*R* = 1.00).

**TABLE 4:** Effect size estimate, standard error and confidence intervals produced by model averaging of environmental and behavioural predictor variables on proportion of fledging success in little penguins at Phillip Island, Australia (*N* = 12 breeding seasons).

Parameter	Estimate	Unconditional SE	Confidence interval	Relative importance
*Intercept*	8.508	1.644	(5.286, 11.730)	
Divorce rate	−3.707	0.790	(−5.256, −2.159)	1.00
Foraging trip duration (days)	−3.616	0.689	(−4.966, −2.266)	1.00
SOI	−0.032	0.009	(−0.051, −0.015)	1.00
SST	0.173	0.184	(−0.188, 0.534)	0.08

### 3.4 Number of Fledglings

**FIGURE 4:**
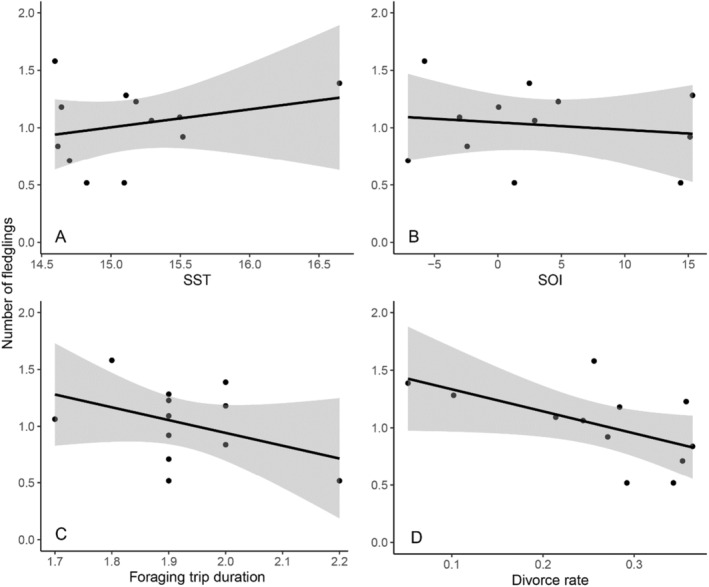
Average number of fledglings by pairs each breeding season (2001–2012) in a population of little penguins, at Phillip Island, Australia, against predictor variables (A) SST, (B) SOI, (C) foraging trip duration (days) and (D) divorce rate.

The average number of fledglings per pair each season varied across the monitoring period (average 1.03, range 0.52–1.58, Figure S1d). However, the effect sizes and confidence intervals for all predictor variables indicated no statistically significant relationships with the number of fledglings (Table 5, Table S5, Figure 4).

**TABLE 5:** Effect size estimate, standard error and confidence intervals produced by model averaging of environmental and behavioural predictor variables on the average number of fledglings produced per pair in little penguins at Phillip Island, Australia (*N* = 12 breeding seasons).

Parameter	Estimate	Unconditional SE	Confidence interval	Relative importance
*Intercept*	1.660	1.795	(−1.859, 5.179)	
Divorce rate	−1.986	1.067	(−4.077, 0.105)	0.57
Foraging trip duration (days)	−1.038	0.876	(−2.754, 0.679)	0.21
SOI	−0.012	0.014	(−0.010, 0.015)	0.14
SST	0.051	0.262	(−0.462, 0.565)	0.12

## 4 Discussion

Over 13 breeding seasons, we investigated how environmental and behavioural factors influence population‐level reproductive success in a colony of little penguins on Phillip Island, Australia. We aimed to determine which of environmental conditions, foraging behaviour or rates of divorce was the most consistent predictor of reproductive success. Our hypothesis that shorter average foraging trip durations and lower rates of divorce would lead to increased reproductive success was partly supported. Years with lower divorce rates (i.e., a failure of birds to re‐pair with the partner from the previous season) correlated with higher hatching and fledging success. Longer foraging trip durations during egg incubation increased hatching success of chicks, while in contrast, shorter foraging trips posthatching increased fledging success. This suggests complex dynamics between foraging behaviour and reproductive stages. In years of lower SOI values, there was an increase in both hatching and fledging success, while SST did not influence any measure of reproductive success, thus our hypothesis that better environmental conditions (higher SOI values and lower SST values) would increase reproductive success was not supported. These findings highlight the complexity of reproductive success determinants at different stages and highlight the important roles of social stability and foraging behaviour, particularly divorce rate, as the most consistent predictor of reproductive outcomes in little penguins.

### 4.1 Reduced Divorce Rate Increases Reproductive Success

Divorce rate was the most consistent factor for predicting reproductive success in this population, with seasons of low population‐level divorce rates (i.e., seasons with high retention of the previous season's partner) leading to an increased proportion of successful hatching and fledging. Little penguins and other seabirds that prolong their pair bond over multiple seasons experience increased reproductive success over time (Black 1996; Kim et al. 2007; Nisbet and Dann 2009). However, divorce may also be an adaptive tactic to increase longer term reproductive success (Culina, Radersma, and Sheldon 2015), especially when the previous breeding success was low (Setiawan et al. 2005), a higher‐quality mate becomes available or usurps a lower‐quality individual (Ens, Safriel, and Harris 1993; Choudhury 1995) or environmental events prevent or delay re‐pairing (Gunnarsson et al. 2004; Afán et al. 2015). However, while divorce can improve reproductive output in the long term, it can have a negative effect on reproductive success in the short term (Gousy‐Leblanc et al. 2023), as observed in this population. Divorce in birds can incur various costs, including the effort required to find a new mate, increased conflict with rivals, the risk of pairing with a lower‐quality mate, no breeding familiarity and reduced reproductive efficiency during the early stages of a new partnership (Ens, Choudhury, and Black 1996; Gousy‐Leblanc et al. 2023). These costs have reduced reproductive success in other species (Ismar et al. 2010; Sanchez‐Macouzet, Rodriguez, and Drummond 2014; Gousy‐Leblanc et al. 2023). Newly formed Australasian gannets ( *Morus serrator* ) pairs have a higher chance of reproductive failure compared to reunited pairs (Ismar et al. 2010). Blue‐footed boobies ( *Sula nebouxii* ) that maintained their pair bond establish clutches earlier, have a higher hatching success and produce more fledglings than those birds with unfamiliar mates (Sanchez‐Macouzet, Rodriguez, and Drummond 2014). Thus, maintaining stable pair bonds seems a crucial strategy for maximising immediate reproductive success in seabirds.

### 4.2 Short Foraging Trip Duration Increases Fledging Success, but Reduces Hatching Success

The little penguin population had a higher proportion of fledging success in years with shorter average foraging trip durations, while longer foraging trips increased the proportion of hatching success. Previous studies have shown that shorter foraging trips are correlated with higher fledging success in little penguins and that they are likely associated with good foraging conditions, which permit parents to get food for their chicks more quickly (Chiaradia and Nisbet 2006; Saraux et al. 2011b). These findings are consistent with studies on seabirds in general, where poorer foraging opportunities increase time spent away from chicks, with smaller meal sizes and less frequent meal delivery to chicks resulting in lower reproductive success (Weimerskirch et al. 1997; Chivers et al. 2012; Boersma and Rebstock 2009). Alternatively, during the egg incubation phase, we found that longer foraging trips were associated with increased proportions of hatched eggs within the population. This result contrasts with other studies in poor breeding seasons when longer foraging trips were associated with lower hatching success (Chiaradia and Kerry 1999; Kemp and Dann 2001). If the incubating partner is left a long time to incubate, they may be forced to abandon the nest due to depleted fat stores (Olsson 1997). However, little penguins in this population spent as long as 9 days on foraging trips and still successfully hatched eggs (Kato, Ropert‐Coudert, and Chiaradia 2008) and the longest average foraging trip duration observed in this study was 2.2 days, which is well within the limits that fat stores last in this species (Gales and Green 1990). This suggests food may not have been a limiting factor for adults during the study period and adults may have been able to forage to satiation without being limited by needing to return to the nest to allow their partner to forage, thus maintaining optimal body condition (Weimerskirch et al. 1997). Furthermore, less frequent changes of the incubating partner could reduce predation risk by keeping the nest concealed, increasing incubation success (Shoji et al. 2011). Therefore, while foraging trip duration is an important factor mediating success in chick‐rearing, it is not generally a reliable factor in predicting incubation due to conflicting results found across different studies.

### 4.3 Environmental Conditions

Marine environmental conditions had mixed impacts on little penguins' population‐level reproductive success in this study. Our unexpected finding that negative SOI values correlated with increased hatching and fledging success highlights the complex and localised responses of seabird breeding success to large‐scale oceanographic indices. While positive SOI values are often associated with enhanced marine productivity and improved breeding (Berlincourt and Arnould 2015; Chiaradia and Nisbet 2006), the spatial variability and lag effects of environmental changes in the Bass Strait region may influence these dynamics (Jenkins et al. 2022). Similar to the delayed breeding responses observed in small procellariiform seabirds during marine heatwave years (Eizenberg et al. 2021), the relationship between SOI and reproductive success could reflect local environmental conditions, such as prey availability and distribution, that differ from broader oceanographic trends (Jenkins et al. 2022). Further investigation into these localised mechanisms is necessary to understand how SOI interacts with site‐specific factors influencing little penguin breeding. In other seabirds, SST has been shown to influence reproductive success (Chambers 2004; Becker, Peery, and Beissinger 2007; Erwin and Congdon 2007) and affect divorce rates (Ventura et al. 2021). Similarly, we found a negative correlation between SST and divorce rates, but SST did not significantly influence the measures of reproductive success. This suggests that SST should be used with caution as an indicator of population reproductive success, as has been identified previously (Afán et al. 2015). In one population, warmer SST in months before breeding led to more chicks and larger fledglings (Cullen et al. 2009), whereas in another population, the opposite was found (Cannell et al. 2012). Little penguins may be able to mitigate the consequences of local variable environmental conditions by using flexible behaviours. Seabirds exhibit plasticity in their foraging strategies, whereby they alter their foraging spatially, temporally and, in some cases, target different prey species to buffer the effects of unpredictable prey availability (Burke and Montevecchi 2009; Garthe, Montevecchi, and Davoren 2011; Bourgeois et al. 2022).

## 5 Conclusions

Here we highlight the interplay between environmental, behavioural and social factors in determining reproductive success in little penguins. We identify divorce rates as a reliable predictor of reproductive outcomes, with lower divorce rates (higher retention of the previous breeding season's partner) associated with higher reproductive success. Foraging behaviour also plays a significant role, with shorter trips enhancing fledging success and longer trips during incubation boosting hatching success. However, environmental variables, particularly SST and the SOI, showed mixed effects, suggesting that broader oceanographic indices may not always be reliable predictors of reproductive success at the local scale. This research emphasises the need for an integrated approach when studying seabird reproductive success, as individual behavioural strategies and social dynamics can potentially outweigh environmental signals. Our results also suggest that monitoring divorce rates could offer a valuable, noninvasive tool for tracking reproductive trends in seabirds, particularly in populations facing fluctuating environmental conditions.

## Author Contributions

**Matthew D. Simpson:** conceptualization (equal), data curation (lead), formal analysis (supporting), investigation (lead), methodology (equal), writing – original draft (lead), writing – review and editing (supporting). **Ashton L. Dickerson:** data curation (supporting), formal analysis (lead), methodology (supporting), writing – review and editing (lead). **Andre Chiaradia:** conceptualization (equal), data curation (supporting), formal analysis (supporting), funding acquisition (equal), investigation (equal), methodology (equal), project administration (equal), supervision (equal), writing – original draft (supporting), writing – review and editing (supporting). **Lloyd Davis:** conceptualization (equal), formal analysis (supporting), funding acquisition (equal), methodology (equal), writing – original draft (supporting), writing – review and editing (supporting). **Richard D. Reina:** conceptualization (equal), data curation (supporting), formal analysis (supporting), funding acquisition (equal), methodology (equal), supervision (equal), writing – original draft (supporting), writing – review and editing (supporting).

## Conflicts of Interest

The authors declare no conflicts of interest.

## Supporting information


Appendix S1

## Data Availability

The data set generated and analysed during the current study are available in the Figshare repository, https://doi.org/10.6084/m9.figshare.26830978.v1
