# Supplementary material for: Divorce Rates Better Predict Population‐Level Reproductive Success in Little Penguins Than Foraging Behaviour or Environmental Factors
Source: Ecol Evol. 2025 Jan 11;15(1):e70787. doi: 10.1002/ece3.70787 (PMC11724333; doi:10.1002/ece3.70787)
Supplement: Supplementary file 1 — Appendix S1 [file ECE3-15-e70787-s001.docx]

**Table S1.** Description of the different stages within the little penguin breeding season and the general length (days) along with definitions of commonly used terms.

|  |  | **Description** | **Length** | **Reference** |
| --- | --- | --- | --- | --- |
| Breeding stage | *Incubation* | One parent will sit on eggs until hatching occurs, parents alternate but this schedule is not fixed | 35 ± 1 days | (Chiaradia & Kerry, 1999; Schreiber & Burger, 2001) |
|  | *End Incubation* | Incubation ends when chicks hatch |  |  |
|  | *Guard* | Once chicks have hatched one parent will be present, guarding chicks, with the other parent returning every few days to alternate roles | 15-20 days | (Chiaradia & Kerry, 1999; Chiaradia & Nisbet, 2006) |
|  | *End Guard* | The guard stage ends when a parent is no longer present within the nest |  |  |
|  | *Post Guard* | Chicks are left on their own during the day while both parents forage and parents will return each night to feed chicks until they are ready to fledge | Variable  (6-8 weeks) | (Chiaradia & Nisbet, 2006; Saraux *et al.*, 2011a) |

**Table S2.** Average number of eggs by pairs. Greyed out models were removed from the final subset of models for averaging due to identified uninformative parameters following the decision tree of Leroux 2019 and a Likelihood Ratio Test to test if models were significantly improved.

|  | (Intrc) | DR1 | FTD | SOISJ | SSTSJ | df | logLik | AICc | delta | weight |
| --- | --- | --- | --- | --- | --- | --- | --- | --- | --- | --- |
| 1 | 1.927 |  |  |  |  | 2 | 16.288 | -27.2 | 0 | 0.343 |
| 2 | 2.006 | -0.304 |  |  |  | 3 | 17.738 | -26.5 | 0.77 | 0.234 |
| 3 | 1.582 |  | 0.179 |  |  | 3 | 17.001 | -25 | 2.24 | 0.112 |
| 4 | 1.59 | -0.3455 | 0.222 |  |  | 4 | 19.183 | -24.7 | 2.59 | 0.094 |
| 5 | 1.921 |  |  | 0.002008 |  | 3 | 16.657 | -24.3 | 2.93 | 0.079 |
| 6 | 1.601 |  |  |  | 0.02152 | 3 | 16.513 | -24 | 3.22 | 0.069 |
| 7 | 2.465 | -0.4189 |  |  | -0.02833 | 4 | 17.995 | -22.3 | 4.97 | 0.029 |
| 8 | 1.997 | -0.2812 |  | 0.000957 |  | 4 | 17.833 | -22 | 5.29 | 0.024 |
| 9 | 1.561 |  | 0.1864 | 0.002157 |  | 4 | 17.484 | -21.3 | 5.99 | 0.017 |

**Table S3.** Hatching success (proportion of eggs hatched by number of eggs laid). Greyed out models were removed from the final subset of models for averaging due to identified uninformative parameters following the decision tree of Leroux 2019 and a Likelihood Ratio Test to test if models were significantly improved.

|  | (Intrc) | DR1 | FTD | SOISJ | SSTSJ | df | logLik | AICc | delta | weight |
| --- | --- | --- | --- | --- | --- | --- | --- | --- | --- | --- |
| 1 | -0.6199 | -4.265 | 1.641 | -0.02037 |  | 4 | -55.323 | 124.4 | 0 | 0.494 |
| 2 | -0.8735 | -3.764 | 1.662 |  |  | 3 | -58.597 | 126.2 | 1.83 | 0.197 |
| 3 | 4.077 | -5.187 | 1.896 |  | -0.3315 | 4 | -56.739 | 127.2 | 2.83 | 0.12 |
| 4 | 4.052 | -5.509 | 1.84 | -0.0199 | -0.312 | 5 | -53.645 | 127.3 | 2.93 | 0.114 |
| 5 | 2.438 | -3.907 |  | -0.02125 |  | 3 | -59.577 | 128.2 | 3.79 | 0.074 |

**Table S4.** Fledgling success (proportion of chicks fledged out of the number of eggs hatched). All models were used for model averaging as no models containing uninformative parameters were identified.

|  | (Intrc) | DR1 | FTD | SOISJ | SSTSJ | df | logLik | AICc | delta | weight |
| --- | --- | --- | --- | --- | --- | --- | --- | --- | --- | --- |
| 8 | 8.693 | -3.764 | -3.602 | -0.03305 |  | 4 | -76.059 | 165.8 | 0 | 0.924 |
| 16 | 6.239 | -3.01 | -3.792 | -0.03215 | 0.173 | 5 | -75.42 | 170.8 | 5.01 | 0.076 |

**Table S5.** average number of fledglings (per pair). All models were used for model averaging as no models containing uninformative parameters were identified.

|  | (Intrc) | DR1 | FTD | SOISJ | SSTSJ | df | logLik | AICc | delta | weight |
| --- | --- | --- | --- | --- | --- | --- | --- | --- | --- | --- |
| 1 | 1.526 | -1.912 |  |  |  | 3 | -1.077 | 11.2 | 0 | 0.344 |
| 2 | 1.027 |  |  |  |  | 2 | -3.388 | 12.1 | 0.96 | 0.213 |
| 3 | 3.191 |  | -1.125 |  |  | 3 | -2.297 | 13.6 | 2.44 | 0.102 |
| 4 | 1.665 | -2.266 |  | -0.01489 |  | 4 | 0.007 | 13.7 | 2.55 | 0.096 |
| 5 | 3.229 | -1.742 | -0.908 |  |  | 4 | -0.062 | 13.8 | 2.68 | 0.09 |
| 6 | -1.341 |  |  |  | 0.1564 | 3 | -2.934 | 14.9 | 3.71 | 0.054 |
| 7 | 3.754 | -2.47 |  |  | -0.1376 | 4 | -0.814 | 15.3 | 4.19 | 0.042 |
| 8 | 1.047 |  |  | -0.00641 |  | 3 | -3.249 | 15.5 | 4.34 | 0.039 |
| 9 | 0.66 |  | -1.187 |  | 0.1752 | 4 | -1.604 | 16.9 | 5.77 | 0.019 |

**Figure S1.** The reproductive patterns of the little penguins across the study period (2001 – 2012), with a) the average number of eggs laid per pairs monitored, b) the proportion of hatched eggs by eggs laid, c) the proportion of fledged chicks by number of chicks hatched and d) the average number of fledglings by the eggs laid (N breeding seasons = 12).

**Figure S2.** Variation in predictor variables across the study period (2001 – 2012) of environmental variables a) delta temperature (not included in models due to co-linearity with SST), b) sea surface temperature and c) SOI and behavioural variables d) average foraging trip duration and e) divorce rate (N breeding seasons = 12).
